# Whole Genome Sequencing of the Braconid Parasitoid Wasp *Fopius arisanus*, an Important Biocontrol Agent of Pest Tepritid Fruit Flies

**DOI:** 10.1534/g3.117.040741

**Published:** 2017-06-05

**Authors:** Scott M. Geib, Guang Hong Liang, Terence D. Murphy, Sheina B. Sim

**Affiliations:** * United States Department of Agriculture–Agricultural Research Service Daniel K. Inouye US Pacific Basin Agricultural Research Center, Hilo, Hawaii 96720; † Fujian Agriculture and Forestry University, Forestry College, Fujian Province, China; ‡ National Center for Biotechnology Information, National Library of Medicine, National Institutes of Health, Bethesda, Maryland

**Keywords:** whole genome sequencing, braconid wasp, tephritid fruit fly, biocontrol, Genome Report

## Abstract

The braconid wasp *Fopius arisanus* (Sonan) is an important biological control agent of tropical and subtropical pest fruit flies, including two important global pests, the Mediterranean fruit fly (*Ceratitis capitata*), and the oriental fruit fly (*Bactrocera dorsalis*). The goal of this study was to develop foundational genomic resources for this species to provide tools that can be used to answer questions exploring the multitrophic interactions between the host and parasitoid in this important research system. Here, we present a whole genome assembly of *F. arisanus*, derived from a pool of haploid offspring from a single unmated female. The genome is ∼154 Mb in size, with a N50 contig and scaffold size of 51,867 bp and 0.98 Mb, respectively. Utilizing existing RNA-Seq data for this species, as well as publicly available peptide sequences from related Hymenoptera, a high quality gene annotation set, which includes 10,991 protein coding genes, was generated. Prior to this assembly submission, no RefSeq proteins were present for this species. Parasitic wasps play an important role in a diverse ecosystem as well as a role in biological control of agricultural pests. This whole genome assembly and annotation data represents the first genome-scale assembly for this species or any closely related Opiine, and are publicly available in the National Center for Biotechnology Information Genome and RefSeq databases, providing a much needed genomic resource for this hymenopteran group.

*Fopius arisanus* is a braconid wasp known to attack several species of destructive tephritid fruit fly pests. This species is predominantly an egg parasitoid, meaning females lay eggs on the eggs and early-instar larvae of its hosts, two of which are the Oriental fruit fly, *Bactrocera dorsalis*, and the Mediterranean fruit fly, *Ceratitis capitata* (Haramoto and Bess 1970). In addition, it has a haplodiploid mating system, with male offspring derived from unfertilized eggs, and females from eggs fertilized by male sperm. Due to its polyphagy and compatibility with mass-rearing, it has been released in fruit-fly-infested areas for biocontrol (Bess *et al.* 1961; Manoukis *et al.* 2011; Vargas *et al.* 2012). Large-scale releases of *F. arisanus* in Hawaii in 1950 and 1951 in French Polynesia in 2002 resulted in high levels of parasitism, and were shown to be effective in reducing the *B. dorsalis* and *C. capitata* infestation in release areas (Bess *et al.* 1961; Clausen *et al.* 1965; Purcell 1998; Vargas *et al.* 2002, 2012).

Though many studies have been conducted on the biology of *F. arisanus* (Wang and Messing 2003; Vargas *et al.* 2002), their efficacy in biocontrol (Clausen *et al.* 1965; Haramoto and Bess 1970; Harris *et al.* 2010), and its ability to be mass-reared for release (Bautista *et al.* 1999; Manoukis *et al.* 2011), there exist few genomic resources for the species. This study presents the first assembly of the *F. arisanus* genome and gene set and complements the previous transcriptome assembly by Calla *et al.* (2015). As *F. arisanus* continues to be reared and released for biocontrol of *B. dorsalis* and *C. capitata*, more research needs to be completed to improve the quality of released individuals. This publicly available genome assembly can now contribute genetic and genomic research that will facilitate in its use as a biocontrol agent.

## Materials and Methods

### Wasp samples

#### Laboratory colonies and rearing conditions:

Specimens used for whole genome sequencing and assembly were derived from the USDA-ARS Pacific Basin Agricultural Research Center insectary line of *F. arisanus*, maintained on *B. dorsalis* as its host. Parasitoids were reared as previously described (Manoukis *et al.* 2011; Bautista *et al.* 1999). In this study, adult *F. arisanus* were collected immediately after ecolsion from pupae and isolated by sex, to ensure collection of unmated males and females. A single generation of single male, single female isolated mating was performed, to generate a pool of sibling wasps. These wasps were again immediately isolated by sex after eclosion to collect virgin females. These unmated virgin females were allowed to lay eggs to generate haploid male offspring. All offspring generated are either full- or half-siblings under this scheme. Unmated female wasps from the F1 generation were placed in small rearing containers containing spun honey and water, and several hundred *B. dorsalis* eggs were exposed to each individual female wasp, allowing it to parasitize with unfertilized eggs. Host *B. dorsalis* eggs were allowed to hatch and develop in standard diet until reaching pupal stage. At that stage, pupae were collected, placed in an isolation grid system, and eclosing adults were collected, and immediately frozen in liquid nitrogen and stored at −80°. All resulting progeny were male, which served to verify that females were unmated and progeny will be haploid male. The progeny from the most productive two females were independently used for subsequent whole genome sequencing.

#### DNA extraction methods:

DNA extraction was performed independently on the haploid adult males *F. arisanus* collected as described above. Individual whole wasps were homogenized in tissue lysis buffer using a FastPrep 24 homogenizer (MP Biomedicals, Santa Ana, CA) for 20 sec at 4 min/sec. Homogenized samples were incubated in a 55° water bath for 3 hr, followed by DNA extraction on a Kingfisher Flex 96 automated extraction instrument (Thermo Scientific, Waltham, MA), using standard protocols, and a Mag-Bind Tissue DNA KF Kit (Omega Bio-Tek, Norcross, GA). The quantity and quality of the extracted DNA sample was determined using the High Sensitivity Genomic DNA Analysis Kit on a Fragment Analyzer (Advanced Analytical, Ankeny, IA).

### Whole genome sequencing, assembly, and analysis

#### Library construction, sequencing, and assembly:

Library preparation methods were used to optimize genome assembly with the ALLPATHS-LG assembler. A 180 bp insert Illumina TruSeq fragment library was constructed from 500 ng of DNA extracted from a single haploid male wasp (specimen USDA ID: 24.2, NCBI BioSample: SAMN03010499, the second male from the 24th isolated mating). An Illumina Nextera 3 kb mate-pair library was generated using male haploid siblings of this individual (Pool USDA ID: 24.pool, NCBI BioSample:SAMN03010500), and a pool of half-sibs from a different female (USDA-ID: 12.pool, NCBI BioSample:SAMN03010501) were used to generate an 8 kb mate-pair library, due to limited amount of DNA from the first sibling pool. A single BioSample describing all samples together is presented as NCBI BioSample:SAMN03083650, associated with BioProject:PRJNA258104. The fragment and mate-paired libraries were sequenced using 2 × 100 bp paired-end sequencing on Illumina HiSeq 2500 in High Output mode.

Raw reads from the fragment and mate pair libraries were used to construct a scaffold assembly using ALLPATHS-LG (v.49856) (Gnerre *et al.* 2011) with default parameters with the exception of addition of ”HAPLOIDIFY = TRUE.” Kmer based error correction of the fragment library was performed prior to assembly as part of the ALLPATHS-LG pipeline.

#### Genome annotation and analysis:

Structural and functional annotation of genes was performed with the NCBI Eukaryotic Genome Annotation Pipeline. This automated annotation pipeline utilized transcript evidence from existing RNA-seq data for *F. arisanus* (Calla *et al.* 2015) in addition to NCBI RefSeq protein sets for *Microplitis demolitor*, *Drosophila melanogaster*, *Apis melifera*, and *Nasonia vitripennis*, and 78,705 NCBI GenBank Insecta proteins aligned to the genome to inform gene model prediction using the NCBI eukaryotic gene prediction tool *GNOMON*. Prior to this study, there were no RefSeq proteins for this species curated in NCBI. An overview of the annotation release (*F. arisanus* annotation release 100) is available online at https://www.ncbi.nlm.nih.gov/genome/annotation_euk/Fopius_arisanus/100/.

**Figure 1:**
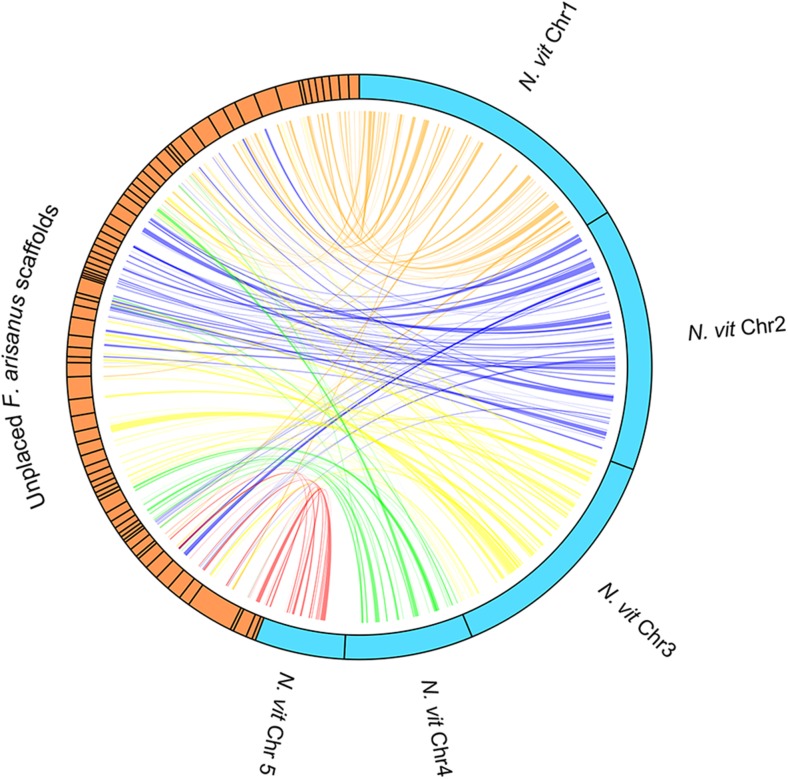
Collinear gene blocks between *F. arisanus* and *N. vitripennis*. Scaffolds from the *F. arisanus* assembly containing collinear orthologous gene blocks which consist of three or more genes in the same order in as the chromosome assembly of the *N. vitripennis* genome. The assembled chromosomes of *N. vitripennis* are represented as turquoise bars and the *F. arisanus* scaffolds are represented by orange bars. The links between the collinear blocks between the *F. arisanus* and *N. vitripennis* assemblies are colored by the chromosome in which they are located in the *N. vitripennis genome* (links to the *N. vitripennis* chromosome 1 are in orange, links to chromosome 2 are in blue, links to chromosome 3 are in yellow, links to chromosome 4 are in green, and links to chromosome 5 are in red).

The completeness of the genome and gene set was analyzed by identifying the number of arthropod Benchmark Universal Single-Copy Orthologs (BUSCOs) (Simão *et al.* 2015). BUSCO v1.1b1 was run on both the RefSeq Gene Set at the predicted peptide level (using ”-m OGS”), and also directly on the scaffolded genome assembly (using ”-m genome”). In addition, this assembly was compared to the *Nasonia vitripennis*, currently one of the most complete parasitoid hymenopteran genomes available, with a large portion of the genome being mapped to one of five chromosomes (Werren *et al.* 2010). Putative orthologs between the two genomes were identified through reciprocal blast between the *F. arisanus* and *N. vitripennis* RefSeq peptide sets. These blast results were analyzed with MCScanx (Wang *et al.* 2012) to identify collinear gene blocks consisting of three or more genes that are in the same linear order in *F. arisanus* as they are in *N. vitripennis*. Links between the identified collinear gene blocks in both species were visualized using RCircos (Zhang *et al.* 2013) (Figure 1).

### Data availability

All raw sequencing data and the assembly is associated with NCBI BioProject: PRJNA258104 within GenBank and PRJNA274979 within RefSeq. This includes three SRA accessions, SRX689044 (180 bp fragment data, described in NCBI BioSample SAMN03010499), SRX689045 (3 kb mate-pair data, described in NCBI BioSample SAMN03010500), and SRX689047 (8 kb mate-pair data, described in NCBI BioSample SAMN03010501). The genome assembly, WGS Project JRKH01, is represented as BioSample SAMN03083650 and is present in GenBank as assembly accession GCA_000806365.1 and RefSeq as assembly accession GCF_000806365.1 (they are identical) named ASM80636v1. All structural annotations are associated with the RefSeq assembly accession, and are considered annotation release 100. An FTP site for data download is at ftp://ftp.ncbi.nlm.nih.gov/genomes/Fopius_arisanus/, a species-specific BLAST page at https://www.ncbi.nlm.nih.gov/genome/seq/BlastGen/BlastGen.cgi?taxid=64838, NCBI’s Genome Data Viewer at https://www.ncbi.nlm.nih.gov/genome/gdv/?org=fopius-arisanus and overview of release 100 annotations at https://www.ncbi.nlm.nih.gov/genome/annotation_euk/Fopius_arisanus/100/. Curation of this assembly, in addition to NCBI, is hosted by the USDA National Agricultural Library I5K workspace (https://i5k.nal.usda.gov/) allowing for visualization within JBrowse, manual curation, and other tools.

## Results and Discussion

In total, ∼21.2 Gb of fragment library (∼137× coverage), 17.9 Gb of 3 kb mate-pair data (∼116× coverage), and 2.7 Gb of 8 kb mate-paired data (∼17.5× coverage) was collected (Table 1).

**Table 1:** Raw reads generated for assembly

SRA	Library Type	Read Pairs	Base Pairs	Coverage
SRX689044	180 bp	106.1 M	21.2 Gb	137×
SRX689045	3 kb	89.7 M	17.9 Gb	116×
SRX689047	8 kb	21.0 M	4.2 Gb	17.5×

Assembling this data with ALLPATHS-LG yielded a fairly contiguous assembly, containing 8510 contigs with an N50 contig size of 51,867 (including contigs >1 kb) placed onto 1042 scaffolds, with an N50 scaffold size of 0.978 Mb. This assembly is 153.63 Mb in length, with only 8.2% of the genome in scaffold gaps and half of the genome placed into 49 scaffolds with a GC content of 38.6% (Table 2). There are three additional braconid genomes in the NCBI WGS database, (*Diachasma alloeum*, *Microplitis demolitor*, and *Cotesia vestalis* (*C. vestalis* is not scaffolded). Assembly statistic of *F. arisanus* are compared to those species as well as the model parasitoid *N. vitripennis* in Table 2, showing very similar GC content and similar genome sizes between species.

**Table 2:** Assembly summary statistics compared to other parasitoid genomes

Species	NCBI Bio Project (PR-JNA#)	Contig Count (N50 kb)	Scaffold Count (N50 Mb)	Total Length (Mb)	GC (%)
*F. arisanus*	258104	8510 (51.90)	1042 (0.98)	153.6	39.4
*N. vitripennis*	13660	25484 (18.84)	6169 (0.71)	295.8	40.6
*D. alloeum*	306876	25534 (44.93)	3968 (0.65)	388.8	39.1
*M. demolitor*	195937	27508 (14.12)	1794 (1.14)	241.2	33.1
*C. vestalis*	271135	9156 (46.06)	—	186.1	30.6

Genome annotation through the NCBI Eukaryotic Annotation Pipeline annotated 11,691 genes or pseudogenes, with 10,991 being protein coding gene regions. In total, 18,906 transcripts across these genes were annotated, with 3826 genes having more than one transcript variant identified. Evidence for the gene annotations was derived from either evidence (*e.g.*, RNA-Seq data or proteins from related species) or from *ab initio* evidence predicted by GNOMON. A large proportion of the transcripts (mRNAs) were fully supported from experimental evidence, with 17,854 of the 18,906 (94.4%), showing the utility of high-quality RNA-Seq data to support gene annotation. In addition to protein coding genes, 670 noncoding genes were identified, including tRNA, lncRNA, and others. Details of the annotation are present in Table 3, as well as on-line at https://www.ncbi.nlm.nih.gov/genome/annotation_euk/Fopius_arisanus/100/.

**Table 3:** Gene annotation summary statistics

Feature	Count	Mean Length (bp)	Median Length (bp)	Minimum Length (bp)	Maximum Length (bp)
Genes	11,661	8569	3152	71	490,550
All transcripts	20,216	2844	2143	71	53,694
mRNA	18,906	2947	2228	248	53,694
misc RNA	367	2687	2071	174	13,135
tRNA	159	74	73	71	84
lncRNA	784	996	785	106	7,102
CDSs	18,906	1964	1419	105	52,947
Exons	71,080	442	216	2	14,501
Introns	57,960	1625	214	30	332,337

An assessment of the completeness of the genome based off of presence of core Insecta genes utilizing BUSCO, suggested a very complete annotation set, with 97% of the Insecta BUSCOs being present within the RefSeq annotation set, and only 1.3% of those fragmented. In comparison, running BUSCO analysis on a previously published RNA-Seq assembly for this species (Calla *et al.* 2015), the BUSCO completeness was only 67% with 5.6% of those fragmented, despite attempts to include as many life stages and samples into the RNA-Seq assembly. Comparing to the NCBI v102 annotation release for *N. vitripennis*, this annotation set is very comparable, with *N. vitripennis* having 96% of the Arthropoda BUSCOs and 1.4% fragmented. In addition, more recent braconid genomes in NCBI have similar BUSCO results. Details of BUSCO analysis of the RefSeq gene set, genomic scaffolds, and the previously published transcriptome assembly are presented in Table 4, including comparison to other braconid genomes public in NCBI (*Diachasma alloeum* and Microplitis demolitor (note, *Cotesia vestalis* does not have structural annotations) and the model parasitoid *N. vitripennis*.

**Table 4:** BUSCO analysis on assembly and annotations

Species	CDS Count	BUSCO Mode	Complete*^a^*	Fragmented	Missing
*F. arisanus*	18,906	OGS	2605 (97)	37 (1.3)	33 (1.2)
*F. arisanus*	—	Genome	2355 (88)	232 (8.6)	88 (3.2)
*F. arisanus**^b^*	15,346	Trans	1803 (67)	152 (5.6)	720 (26)
*N. vitripennis**^c^*	24,846	OGS	2585 (96)	40 (1.4)	50 (1.8)
*D. alloeum**^d^*	19,692	OGS	2622 (98)	31 (1.1)	22 (0.8)
*M. demolitor**^e^*	18,586	OGS	2621 (97)	34 (1.2)	20 (0.7)

a Number of BUSCO proteins (percent of total BUSCOs).

b From NCBI TSA PRJNA259570.

c From NCBI RefSeq v201 annotation release.

d From NCBI RefSeq v100 annotation release.

e From NCBI RefSeq v101 annotation release.

The development of thoughtful experimental design was critical in the assembly of this genome. One major obstacle of genome assembly is the necessity of minimizing the amount of heterozygosity in the input DNA (Baker 2012). By performing an isolated mating, and subsequent unmated female oviposition, all individuals included in the assembly were haploid. The fragment library, generated from a single haploid individual, and the 3 kb mate-paired library, generated from a pool of haploid brothers of the single individual, approximated the single diploid genome of the parental mother, with the exception of unique recombination events occurring within the mother. This allowed extraction of sufficient DNA quantity to generate these libraries through pooling individuals, without introducing additional heterozygosity into the pooled sample. While the 8 kb library was from a different set of siblings, those siblings were from a common isomating, and extremely closely related to the first set of individuals. Also in ALLPATHS-LG, the large jumping libraries are not integrated into the contig assembly process, rather they are used for scaffolding and higher level assembly refinement. Similar approaches can be taken in other hymenopterans, where unmated females lay haploid offspring, to overcome the small size and low DNA yield in these species.

Having genomic resources for this species will allow for investigation of multitrophic interactions within this species and its hosts at a foundational genomic level. As in other parasitic Hymenoptera, this wasp harbors endosymbiotic viruses such as polydnaviruses and nudiviruses to enable the breeching of host defenses and successful development. In some related species, integration of the viral genome into the wasp has been demonstrated (Pichon *et al.* 2015), and with this reference genome, future research will investigate if similar integration events have occurred in this species as well. This species attacks a broad range of host Tephritid flies, and can be successfully reared on hosts from across several genera. The genetic basis for behavior, host preference, host selection, and competition can be investigated (Wang and Messing 2008; Harris *et al.* 2007).
